# Thiazolidinedione use and atrial fibrillation in diabetic patients: a meta-analysis

**DOI:** 10.1186/s12872-017-0531-4

**Published:** 2017-04-05

**Authors:** Zhiwei Zhang, Xiaowei Zhang, Panagiotis Korantzopoulos, Konstantinos P. Letsas, Gary Tse, Mengqi Gong, Lei Meng, Guangping Li, Tong Liu

**Affiliations:** 1 Tianjin Key Laboratory of Ionic-Molecular Function of Cardiovascular Disease, Department of Cardiology, Tianjin Institute of Cardiology, Second Hospital of Tianjin Medical University, No. 23 Pingjiang Road, Hexi District, Tianjin, 300211 People’s Republic of China; 2 First Department of Cardiology, University of Ioannina Medical School, Ioannina, Greece; 3 Second Department of Cardiology, Laboratory of Cardiac Electrophysiology, “Evangelismos” General Hospital of Athens, Athens, Greece; 4 Department of Medicine and Therapeutics, Faculty of Medicine, Chinese University of Hong Kong, Hong Kong, SAR People’s Republic of China; 5 Li KaShing Institute of Health Sciences, Faculty of Medicine, Chinese University of Hong Kong, Hong Kong, SAR People’s Republic of China

**Keywords:** Atrial fibrillation, Diabetes mellitus, Thiazolidinediones, Pioglitazone, Rosiglitazone, Meta-analysis

## Abstract

**Background:**

Accumulating evidence suggests that thiazolidinediones (TZDs) may exert protective effects in atrial fibrillation (AF). The present meta-analysis investigated the association between TZD use and the incidence of AF in diabetic patients.

**Methods:**

Electronic databases were searched until December 2016. Of the 346 initially identified records, 3 randomized clinical trials (RCTs) and 4 observational studies with 130,854 diabetic patients were included in the final analysis.

**Results:**

Pooled analysis of the included studies demonstrated that patients treated with TZDs had approximately 30% lower risk of developing AF compared to controls [odds ratio (OR): 0.73, 95% confidence interval (CI): 0.62 to 0.87, *p* = 0.0003]. This association was consistently observed for both new onset AF (OR =0.77, *p* = 0.002) and recurrent AF (OR =0.41, *p* = 0.002), pioglitazone use (OR =0.56, *p* = 0.04) but not rosiglitazone use (OR =0.78, *p* = 0.12). The association between TZD use and AF incidence was not significant in the pooled analysis of three RCTs (OR =0.77, 95% CI = 0.53–1.12, *p* = 0.17), but was significantly in the pooled analysis of the four observational studies (OR =0.71, *p* = 0.0003).

**Conclusions:**

This meta-analysis suggests that TZDs may confer protection against AF in the setting of diabetes mellitus (DM). This class of drugs can be used as upstream therapy for DM patients to prevent the development of AF. Further large-scale RCTs are needed to determine whether TZDs use could prevent AF in the setting of DM.

## Background

Atrial fibrillation (AF) is the most prevalent arrhythmia observed in clinical practice, and is associated with significant morbidity and mortality in the popuation. The burden of AF increases over time mainly due to an aging population and to the increasing prevalence of cardiovascular comorbidities. However, strategies to predict and prevent AF are not fully effective [1]. Diabetes mellitus (DM) is one of the strongest independent risk factors for AF incidence, conferring an approximate 40% higher risk of subsequent AF development [2, 3]. It also predicts the recurrence of AF following a successful direct current cardioversion [4]. Moreover, DM increases the risk of developing stroke, heart failure, and cardiovascular death in patients with AF [5]. Although the exact pathophysiological mechanisms linking DM and AF remain incompletely elucidated, an increasing body of evidence suggests that inflammation and oxidative stress may play an important role [6–8].

Thiazolidinediones (TZDs), a class of peroxisome proliferator-activated receptor-γ (PPAR-γ) agonists, are among the most potent insulin-sensitizing drugs [9]. Apart from their anti-diabetic activity, TZDs display several pleiotropic effects including anti-inflammatory and antioxidant actions that may have potential benefits for AF prevention [10, 11]. However, inconsistent results have been reported regarding TZDs use and AF incidence [12–18]. In light of such conflicting data, we performed a comprehensive meta-analysis to evaluate the present evidence and investigate whether the use of TZDs confers benefits in preventing AF.

## Methods

This systematic review was conducted according to the Quality of Reports of Meta-Analyses of Randomized Controlled Trials (QUOROM) recommendations [19] and the guidelines of the Meta-analysis of Observational Studies in Epidemiology Group (MOOSE) [20].

### Inclusion criteria

The studies considered for this meta-analysis were either randomized clinical trials (RCTs) or observational studies that investigated the potential effects of TZDs on AF. The inclusion criteria were as follows: *RCTs*: 1) randomized controlled human trials with a parallel design; 2) comparison of TZDs with control; 3) collecting data on new or recurrent AF during follow-up. *Observational Studies*: 1) comparison of TZDs with control; 2) evaluating new or recurrent AF as an outcome. In the studies of interventions with TZDs no limit in the length of follow-up period was set due to the paucity of relevant studies.

### Search strategies

A systematic literature search was performed by two investigators (Z. Z. and X. Z.) using the online databases of PubMed and Embase to identify relevant studies published before December 2016. The following key terms were used: “thiazolidinediones”, “pioglitazone”, “rosiglitazone”, “troglitazone”, and “atrial fibrillation”. Both investigators independently evaluated the search results and identified potential studies for further assessment. Disagreements were resolved by a third reviewer (T. L.).

### Quality assessment and data extraction

As quality scoring in meta-analyses of RCTs and observational studies is controversial, several key points of study quality were assessed according to a critical review checklist of Wynn et al. [21]. The key points of this checklist and quality assessments of included studies are listed in Table 1.

**Table 1:** Quality assessments of included studies

Study, year	Study type	Randomisation Method	Blinding	Eligibility criteria reported	Study Population representative of normal practice	Method of follow-up properly defined	Equal follow-up between groups	Was loss to follow-up reported or explained	Prospective recruitment	Consecutive recruitment
PROactive, 2005 [12]	RCT	Randomised permuted blocks	Double	Yes	Yes	Yes	Yes	Yes	Yes	Yes
Anglade, 2007 [13]	Case control	NA	NA	Yes	Yes	Yes	Yes	No loss to follow-up	No	Yes
RECORD, 2009 [14]	RCT	Randompermuted blocks	None	Yes	Yes	Yes	Yes	Yes	Yes	Yes
Gu, 2011 [15]	Cohort	NA	NA	Yes	Yes	Yes	Yes	No loss to follow-up	Yes	Yes
Chao, 2012 [16]	Case control	NA	NA	Yes	Yes	Yes	Yes	No loss to follow-up	No	Yes
Liu, 2014 [17]	RCT	Computer	Double	Yes	Yes	Yes	Yes	No loss to follow-up	Yes	Yes
Pallisgaard, 2016 [18]	Cohort	NA	NA	Yes	Yes	Yes	Yes	No loss to follow-up	Yes	Yes

*Abbreviations*: *RCT* randomized controlled trial, *NA* not applicable

Two investigators (Z. Z. and X. Z.) independently extracted the relevant data using a pre-defined spreadsheets. The extracted data elements of the meta-analysis included information on the inclusion criteria, publication details, study design, follow-up duration, daily dosage of TZDs, definition of AF, methods of AF detection, baseline patient characteristics, the variables of multivariate model used in observational studies and results. Disagreements were resolved through discussion or consensus with a third reviewer (T. L.).

### Statistical analysis

Results of the AF outcome are expressed as odds ratio (OR) with 95% confidence interval (CI) for each study using generic inverse-variance method. The hazard ratio value using multivariate Cox proportional hazards model in the primary study was directly considered as OR [22]. Raw event numbers were extracted from the RCTs and adjusted effect estimates from the observational studies to calculate the overall effects. Statistical heterogeneity was assessed by the χ^2^ test and quantified by using the I^2^ statistic. An I^2^ > 50% is indicative of at least moderate heterogeneity [23]. A random-effects model was used. Subgroup analyses regarding AF subtypes (new onset AF or recurrent AF), different TZDs (solely pioglitazone or solely rosiglitazone), study designs (RCTs or observational studies), and different follow-up duration (>5 years or ≤5 years) were additionally performed. Sensitivity analysis was done by removing one study at a time and checking the consequent effects on the effect estimate. Publication bias was evaluated using a funnel plot. Two-tailed *p* values of <0.05 were considered statistically significant. The statistical analysis was performed using the Review Manager (RevMan, version 5.3, Copenhagen: The Nordic Cochrane Centre, The Cochrane Collaboration, 2014).

## Results

**Fig. 1:**
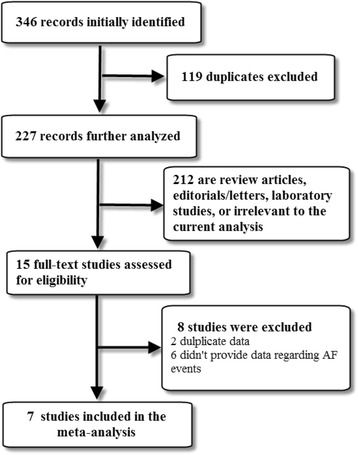
Flow diagram of the study selection process

A total of 346 records were identified initially through our literature search strategy. After careful assessment, seven studies (three RCTs [12, 14, 17] and four observational studies [13, 15, 16, 18]) comprising 130,854 diabetic patients (11,781 in the treatment and 119,073 in the control group) were included in the final meta-analysis (Fig. 1).

Three studies [12, 15, 17] examined the relationship between pioglitazone use and AF, while two other [14, 16] studied rosiglitazone use. The remaining two studies [13, 18] reported data regarding the use of pioglitazone, rosiglitazone and troglitazone. The characteristics of each study are listed in Table 2, and the patients’ characteristics in each study are shown in Table 3.

**Table 2:** The characteristics of 7 included studies

Study, year	Study population	Patients (*n*)	Comparators	Daily dosage of TZDs	Follow-up	Definition of AF	Methods of AF detection	The variables of multivariate model
PROactive, 2005 [12]	Patients with type 2 diabetes who had evidence of macrovascular disease	5238	Pioglitazone (*n* = 2605) vs. placebo (*n* = 2633)	Titrated from 15 to 45 mg	34.5 months	New-onset AF	NA	NA
Anglade, 2007 [13]	Diabetic patients who underwent CABG and/or valvular surgery	184	Pioglitazone (*n* = 14), rosiglitazone (*n* = 24) and troglitazone (*n* = 2) vs. No TZD (*n* = 140)	Pioglitazone: average 30 mg Rosiglitazone: average 6 mg, Troglitazone: average 525 mg	30 days	Postoperative AF	NA	NA
RECORD, 2009 [14]	Patients with type 2 diabetes	4447	Rosiglitazone + metformin or sulfonylurea (*n* = 2220) vs. metformin and sulfonylurea (*n* = 2227)	Titrated from 4 to 8 mg	5.5 years	New-onset AF	NA	NA
Gu, 2011	Type 2 diabetic patients with paroxysmal AF undergoing catheter ablation	161	Pioglitazone (*n* = 51) vs. No pioglitazone (*n* = 99)	30 mg	22.9 ± 5.1 months	Recurrent ATa (AF, AT, AFL)	ECG and Holter recording	Duration of PAF, LAD, treatment with ACEI/ARB
Chao, 2012 [16]	Patients with non-insulin dependent diabetes.	12,065	Rosiglitazone (*n* = 4137) vs. No rosiglitazone (*n* = 7928)	NA	63 ± 25 months	New-onset AF	NA	Age, HTN, CAD, chronic renal disease and use of statins or alpha-glucosidase inhibitors
Liu, 2014 [17]	Diabetic patients with the first presence of persistent AF	146	Pioglitazone (*n* = 70) vs. placebo (*n* = 76)	30 mg	20.1 months	Recurrent AF	ECG, history of arrhythmia-related symptoms, and Holter monitoring	NA
Pallisgaard, 2016 [18]	Diabetic patients of Danish nationwide registries	108,624	TZD (*n* = 2658) vs. other second-line antidiabetic drugs (*n* = 105,966)	NA	12 years	New-onset AF	NA	Age, sex, stroke, HF, all cancer, hyperthyroidism, IHD, COPD, CKD, liver disease, vascular disease, HTN, statin use, prior CABG, and prior PCI

*Abbreviations*: *AF* atrial fibrillation, *PAF* paroxysmal atrial fibrillation, *ATa* atrial tachyarrhythmias, *AT* atrial tachycardia, *AFL* atrial flutter, *ECG* electrocardiograph, *CABG* coronary artery bypass graft, *TZDs* thiazolidinediones, *LAD* left atrial diameter, *ACEI* angiotensin converting enzyme inhibitor, *ARB* angiotensin receptor blocker, *HTN* hypertension, *CAD* coronary arterial disease, *IHD* ischaemic heart disease, *COPD* chronic obstructive pulmonary disease, *CKD* chronic kidney disease, *PCI* percutaneous coronary intervention, *NA* not applicable

**Table 3:** Patients characteristics of 7 included studies

Study, year	Design	Age (years) T/C	Male T/C	HF T/C	HTN T/C	CAD T/C	HbA1c (%) T/C	β-blocker T/C	CCB T/C	ACEI/ARB T/C	Statin T/C	Insulin T/C
PROactive, 2005 [12]	RCT	61.9/61.6	67%/66%	NA	75%/76%	48%/48%	7.8/7.9	55%/54%	34%/37%	70%/70%	43%/43%	33.2%/34%
Anglade, 2007 [13]	Nested case control study of patients from the AFIST I, II and III trials	65.8/67.2	72.5%/71.5%	15.0%/18.8%	90.0%/75.7%	NA	NA	75.0%/75.0%	12.5%/21.5%	75.0%/56.9%	77.5%/61.8%	NA
RECORD, 2009 [14]	RCT	58.4/58.5	51.4%/51.7%	0.5%/0.4%	NA	NA	7.9/7.9	22.6%/20.9%	19.1%/21.6%	43.1%/42.1%	18%/19.2%	NA
Gu, 2011	Prospective cohort study	59.6/58.7	52.9%/45.5%	0/0	62.7%/72.7%	5.9%/5.1%	6.2/6.4	35.3%/37.4%	35.3%/28.3%	56.9%/45.5%	13.7%/12.1%	3.9%/2.0%
Chao, 2012 [16]	Nested case control study of patients from NHIRD	53.7/54.1	52.9%/53.6%	4.1%/4.7%	38.1%/44.5%	16.9%/18.4%	NA	45.5%/46.4%	NA	68.6%/68.3%	59%/57.4%	0/0
Liu, 2014 [17]	RCT	60.70/62.25	74.3%/ 65.8%	0/0	28.6%/30.3%	28.6%/30.3%	6.41/6.19	41.4%/38.2%	20%/17.1%	NA	31.4%/34.2%	NA
Pallisgaard, 2016 [18]	Prospective cohort study	59.59/62.40	56.7%/ 58.1%	2.3%/4.9%	50.2%/48.4%	NA	NA	31.5%/31.5%	NA	58.8%/55.9%	58.0%/53.0%	NA

*Abbreviations*: *RCT* randomized controlled trial, *HF* heart failure, *HTN* hypertension, *CAD* Coronary arterial disease, *HbA1c* haemoglobin A1c, *CCB* calcium channel blocker, *ACEI* angiotensin converting enzyme inhibitor, *ARB* angiotensin receptor blocker, *T/C* thiazolidinediones group/control group, *NA* not applicable

**Fig. 2:**
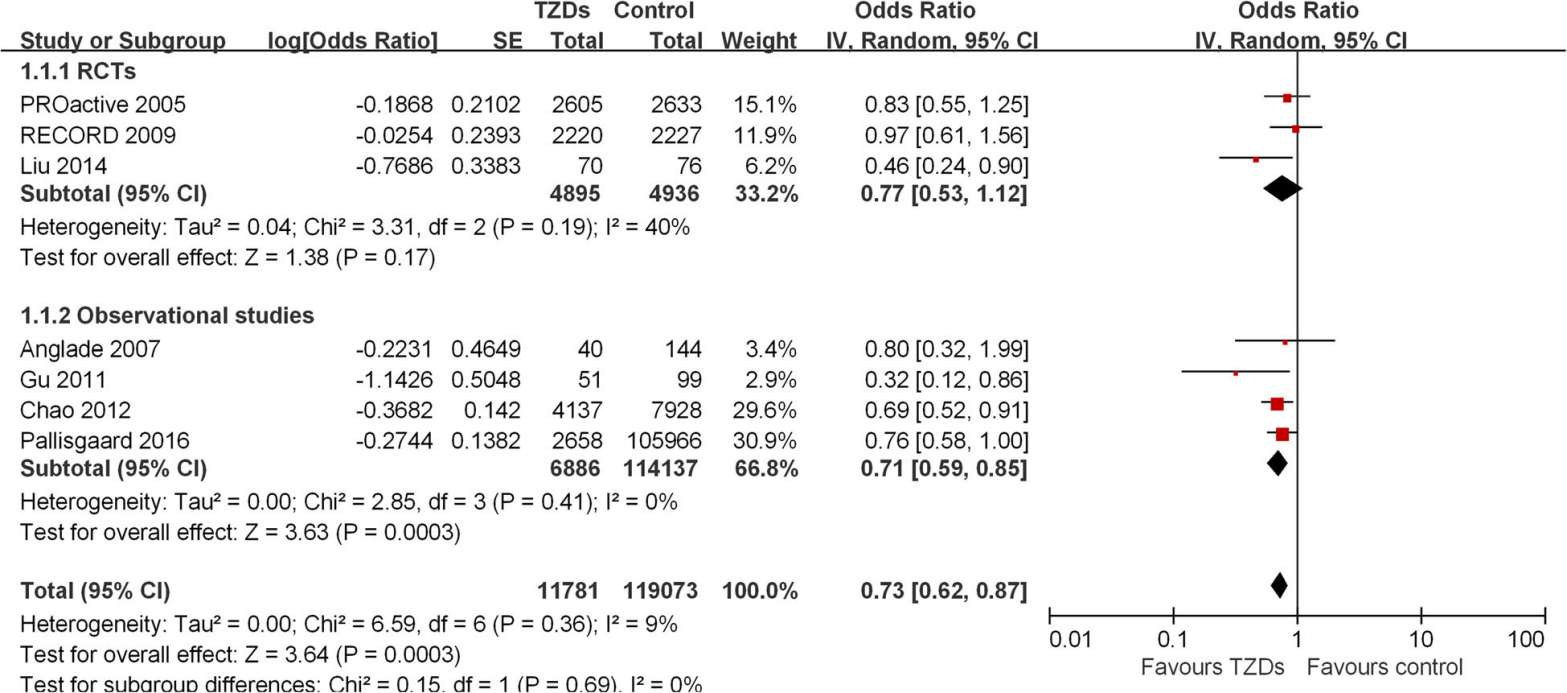
Forest plot showing the association association between thiazolidinediones (TZDs) and atrial fibrillation (AF)

Of the seven studies, four [15–18] studies showed that TZDs use attenuated either the risk of new-onset or recurrent AF, whereas the other three [12–14] studies did not indicate a statistically significant difference. Overall, the pooled analysis of the seven included studies suggested that patients treated with TZDs have nearly 30% lower risk of AF compared with controls (OR =0.73, 95% CI = 0.62–0.87, *p* = 0.0003; Fig. 2). No significant heterogeneity between the individual studies was observed (*P* = 0.36, I^2^ = 9%).

Subgroup analyses according to AF types, different TZDs, follow-up duration, and study designs were subsequently performed (Fig. 2, Table 4). TZDs use was associated with a decrease in the risk of both new-onset [12, 14, 16, 18] (OR =0.77, 95% CI = 0.65–0.91, *p* = 0.002) and recurrent AF [13, 15, 17] (OR =0.41, 95% CI = 0.24–0.72, 0.002) without any heterogeneity across the studies. Regarding different TZDs, pioglitazone use [12, 15, 17] (OR =0.56, 95% CI = 0.32–0.98, *p* = 0.04; I^2^ = 54%) was associated with a lower risk of AF incidence, whereas rosiglitazone use [14, 16] was not significantly associated with a decreasing AF incidence (OR =0.78, 95% CI = 0.57–1.07, *p* = 0.12; I^2^ = 34%). Regarding the subgroup analysis on different follow-up duration, there was no significant difference between the 3 studies [14, 16, 18] with a follow-up duration >5 years (OR =0.76, 95% CI = 0.63–0.91, *p* = 0.002; I^2^ = 0%) and the 4 studies [12, 13, 15, 17] with a follow-up duration ≤5 years (OR =0.62, 95% CI = 0.41–0.94, *p* = 0.02; I^2^ = 34%). Finally, the pooled analysis of the 4 [13, 15, 16, 18] observational studies showed a strong association between TZDs use and risk reduction of AF (OR =0.71, 95% CI = 0.59–0.85, *p* = 0.0003; I^2^ = 0%), whereas the pooled analysis of the three RCTs showed a non-statistically significant 23% reduction in the odds of developing AF (OR =0.77, 95% CI = 0.53–1.12, *p* = 0.10; I^2^ = 40%).

**Table 4:** Subgroup analyses of the association between TZDs and AF

Subgroup	Study	Number of studies	Heterogeneity		Meta-analysis		
			I^2^	*P*-Value	OR	95% CI	*p*-Value
AF types	New-onset AF	4	0%	0.64	0.77	0.65–0.91	0.002
	Recurrent AF	2	0%	0.54	0.41	0.24–0.72	0.002
TZDs	Solely pioglitazone	3	54%	0.11	0.56	0.32–0.98	0.04
	Solely rosiglitazone	2	34%	0.22	0.78	0.57–1.07	0.12
Follow-up duration	≤ 5 years	4	34%	0.21	0.62	0.41–0.94	0.02
	> 5 years	3	0	0.47	0.76	0.63–0.91	0.002
Study design	RCTs	3	40%	0.10	0.77	0.53–1.12	0.17
	Observational studies	4	0%	0.41	0.71	0.59–0.85	0.0003

*Abbreviations*: *TZDs* thiazolidinediones, *AF* atrial fibrillation, *RCTs* randomized controlled trials, *OR* odds ratio, *CI* confidence interval

Besides, due to different pathophysiologic mechanisms of AF, a sensitivity analysis was performed by removing the studies evaluated post-operation AF [13] and post-AF [15] ablation recurrences, no significant differences were found in the heterogeneity (*P* = 0.44; I^2^ = 0%) among the remaining five studies [12, 14, 16–18], and the overall outcome remained the same (OR =0.75, 95% CI = 0.64–0.88, *p* = 0.0003).

## Discussion

The main findings of this comprehensive meta-analysis on 130,854 diabetic patients are the following: i. TZDs may confer protection against AF incidence; ii. the beneficial effects of TZDs were consistently observed in both new onset and recurrent AF; iii. Pioglitazone use was associated with a statistically reduced risk of incident AF, whereas rosiglitazone use showed no statistically significant difference; and iv. the protective effects of TZDs were only observed in the pooled analysis of the observational studies rather than the RCTs.

The PROactive [12] and RECORD [14] RCTs showed that pioglitazone or rosiglitazone use does not provide any benefit in preventing AF incidence among high-risk patients with type 2 DM. However, in these two RCTs, AF was reported as an adverse event rather than a predefined endpoint. Furthermore, these trials displayed a very low AF incidence in both intervention and control groups (1.5–2%), and thus AF detection may be underpowered.

Moreover, in the present meta-analysis, we observed that pioglitazone use was associated with beneficial effects on AF prevention compared with rosiglitazone use. Similarly, previous study suggested that pioglitazone has a beneficial effect on cardiovascular disease, whereas rosiglitazone seemed to increase cardiovascular risk [24]. By assembling a diabetic cohort of older than 65 years, Winkelmayer et al. [25] demonstrated greater risk of mortality and congestive heart failure among patients who initiated therapy with rosiglitazone compared with pioglitazone, however, there were no differences in their incidences of myocardial infarction or stroke. Previous data [26] also showed similar effects on glycemic control between pioglitazone and rosiglitazone, as well as on other parameters such as C-reactive protein (CRP), plasminogen activator inhibitor-1 and indices of insulin secretion and sensitivity. However, pioglitazone treatment was associated with greater beneficial changes on plasma lipids than rosiglitazone treatment [26], which may partly explain the advantage of pioglitazone in reducing AF incidence.

Recently, the IRIS trial [27] demonstrated that pioglitazone can prevent fatal or nonfatal stroke or myocardial infarction among patients who have insulin resistance along with cerebrovascular disease. However, the underlying mechanism for these beneficial effects of pioglitazone remains incompletely elucidated. AF is a known risk factor of morbidity and mortality by predisposing to strokes and acute coronary syndrome [28]. Thus, it is possible to postulate that pioglitazone reduces the stroke or MI events partly through the reduction of AF burden.

Accumulating evidence supports the role of inflammation and immune response activation in the genesis and perpetuation of AF in different clinical settings, including cardiac surgery, electrical cardioversion and catheter ablation [29]. Oxidative stress has been suggested to play an important role in AF incidence [30]. Numerous studies have demonstrated that TZDs may attenuate inflammation and oxidative stress as well as atrial electrophysiological and structural remodeling in different animal models.

In a ventricular tachypacing-induced CHF rabbit model, Shimano et al. [31] showed that pioglitazone prevents atrial structural remodeling and inhibits AF promotion. Also, similarly to candesartan, pioglitazone suppresses transforming growth factor-β1 (TGF-β1) and tumor necrosis factor-α (TNF-α) expression in atrial tissue, molecules that are inflammatory mediators related to fibrosis-mediated AF incidence [29]. More recently, Kume et al. [32] suggested that pioglitazone effectively attenuates inflammatory profibrotic signals and vulnerability to AF in a pressure overload AF rat model, possibly via its suppression in monocyte chemoattractant protein (MCP-1) expression. PPAR-γ agonists have been shown to attenuate Angiotensin II (Ang II) -induced atrial electrical and structural remodeling in cellular models [33]. These effects are mediated by prevention of ICa-L remodeling by inhibiting CAMP responsive element binding protein (CREB) phosphorylation, as well as by suppression of connective tissue growth factor (CTGF) expression and cell proliferation via inhibiting TGF-β1/Smad2/3 and TGF-β1/tumor necrosis factor receptor associated factor 6 (TRAF6)/TGF-β-associated kinase 1 (TAK1) signaling pathways. In addition, Pioglitazone exhibits beneficial effects on Ang II-induced potassium channel remodeling [34]. More recently, Chen et al. [35] further indicated that pioglitazone inhibits Ang II-induced atrial fibroblasts proliferation through nuclear factor-κB (NF-κB)/TGF-β1/Toll/IL-1 receptor domain-containing adaptor inducing IFN-β (TRIF)/TRAF6 signaling pathway. Additionally, Xu et al. [36] suggested that pioglitazone prevents age-related arrhythmogenic atrial remodeling and AF incidence by improving heat shock protein (HSP) 70 expression and antioxidant capacity, and by inhibiting the mitochondrial apoptotic signaling pathway. In an alloxan-induced diabetic rabbit model, we have shown that rosiglitazone attenuates arrhythmogenic atrial structural remodeling and AF incidence via anti-inflammatory and antioxidant effects [37]. In keeping with these findings, the IRIS trial found lower CRP levels in the pioglitazone group than in the placebo group. Indeed, increased CRP levels have been associated with greater risk of AF [38].

Finally, the treatment of hyperglycemia may have favorable effects on AF burden. In other words, treatment of DM may ameliorate atrial remodeling [7]. Haemoglobin A1c levels have been associated with the occurrence and recurrence of AF [7, 39, 40], and therefore TZDs may exert their favorable effects through HbA1c level reduction.

### Study limitations

**Fig. 3:**
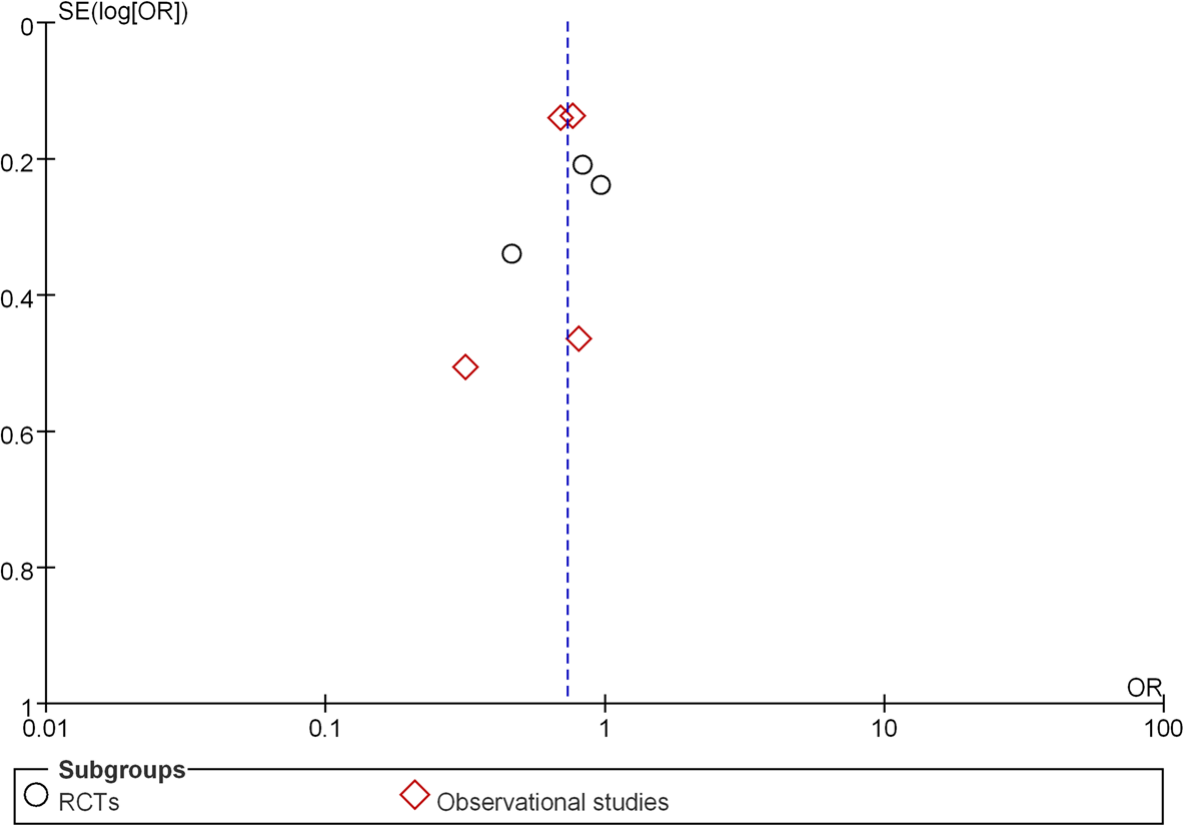
Funnel plot of meta-analysis

The present meta-analysis has potential limitations. Firstly, due to the small number of included studies we analyzed observational studies and RCTs together while 2 included RCTs reported AF as an adverse event rather than a predefined endpoint, and the favorable effects of TZDs use on preventing AF incidence were predominately driven by observational studies, whereas data from the 2 RCTs were unable to draw unanimous conclusion. Secondly, information regarding methods of AF detection, cardiac substrate, ejection fraction and atrial volume were not fully presented in our analysis due to the lack of relative data. Thirdly, the heterogeneous types of patient populations (ranging from uncomplicated type 2 diabetics to post-CABG or post-AF ablation patients) may indicate latent bias in this meta-analysis. Fourthly, “gray” literature (primarily conference abstracts/presentations, ongoing studies, communication with investigators) was not searched. Finally, the results of the funnel plot suggested that publication bias may be present, although the small number of studies made this somewhat difficult to interpret (Fig. 3).

## Conclusions

In summary, this meta-analysis suggests that TZDs may be effective in AF prevention in the setting of DM. Therefore, TZDs may be considered as the treatment of choice in diabetic patient with high risk features for AF incidence. Since the overall conclusion was mainly drawn from the observational studies, further large-scale prospective RCTs that assessed AF as a predefined outcome are needed to determine whether TZDs use could prevent AF in the setting of DM.

## Abbreviations

AF: Atrial fibrillation
CHF: Congestive heart failure
CI: Confidence interval
CRP: C-reactive protein
DM: Diabetes mellitus
OR: Odds ratio
PPAR-γ: Peroxisome proliferator-activated receptor-γ
RCTs: Randomized clinical trials
TZDs: Thiazolidinediones
